# Design and Experiments of a Portable Seabed Integrated Detection Sonar

**DOI:** 10.3390/s21082633

**Published:** 2021-04-09

**Authors:** Jingxin Ma, Haisen Li, Jianjun Zhu, Weidong Du, Chao Xu, Xinyang Wang

**Affiliations:** 1 Acoustic Science and Technology Laboratory, Harbin Engineering University, Harbin 150001, China; majingxin@hrbeu.edu.cn (J.M.); hsli@hrbeu.edu.cn (H.L.); duweidong@hrbeu.edu.cn (W.D.); xuchao18@hrbeu.edu.cn (C.X.); ywj@hrbeu.edu.cn (X.W.); 2 College of Underwater Acoustic Engineering, Harbin Engineering University, Harbin 150001, China; 3 Key Laboratory of Marine Information Acquisition and Security (Harbin Engineering University), Ministry of Industry and Information Technology, Harbin 150001, China

**Keywords:** parametric array, topography detection, sediment geomorphology detection, sub-bottom profile, integrated detection

## Abstract

The integrated observation of seabed topography, sediment geomorphology and sub-bottom profile information is very important for seabed remote sensing and mapping. To improve the efficiency of seabed detection and meet the needs of portable development of detection equipment, we developed a portable seabed feature integrated detection sonar (PSIDS) with whcih a single sonar device can simultaneously detect the above three types of seabed information. The underwater transducer is mainly composed of the following three components: a parametric emission array as the sound source, a high frequency receiving linear array for multibeam echo signal collection, and a two-dimensional vector hydrophone for receiving the low-frequency sediment echo signal. Field experiments were conducted to validate the performance of the PSIDS on 11–17 January 2018 in Jiaozhou Bay, China. (1) PSIDS could perform the functions of both multibeam sonar and sub-bottom profiler; (2) The synchronously and integrated measurement of various seabed information was achieved by alternately emitting multibeam echo-sounding and sub-bottom profiling signal using parametric source. The detection results proved the feasibility and practicability of PSIDS to achieve multiple seafloor characteristics. PSIDS provides a new idea for developing integrated seabed detection sonar. In terms of convenience and data fusion, it is a good option to use this equipment for integrated seabed detection.

## 1. Introduction

In recent years, the importance of seabed resources in the economy is sustained attention. Features information of seabed, such as topography, sediment geomorphology and sedimentary profile, are becoming more and more important in seabed resource surveys and seabed scientific research [1,2,3,4,5,6,7]. At this stage, the seabed detection is mainly based on underwater acoustic technology [8,9,10]. However, no sonar equipment can simultaneously obtain the above three types of feature information, and traditionally, the measurement of the different seabed feature information requires different acoustic detection equipment [11,12,13]. For example, multibeam sounding sonar (MBSS) and side-scan sonar (SSS) can realize the detection of seabed topography and sediment geomorphology, respectively, while sedimentary profile information is generally achieved by sub-bottom profiler (SBP) [14,15,16]. Since the above three sonar devices use independent sound sources, work asynchronous and in different ways, we cannot achieve multiple seabed feature information of the same point at the same time, so it is difficult to realize data fusion and application effectively, many disadvantages are exposed when conducting seabed characteristic detection in this way. First, due to the different installation forms and working principles of the transducers of this equipment (for example, SSS is usually towed behind the surveying ship, while MBSS is fixed to the ship), the attitude information of these devices is different, so it is impossible to observe the same measurement point at the same time. Second, the uncertain factors introduced by time-varying and spatial-varying characteristics of the marine environment make it difficult or impossible to accurately integrate the data measured by multiple instruments [17,18,19]. On the other hand, different sonar equipment and their auxiliary equipment are complicated to install and must be repeatedly calibrated and matched. Especially in deep-sea resource surveying, when taking underwater operation robots as the main carrying platform, due to the limitation of volume, energy and load, it is difficult to install all the above-mentioned sonar devices. Therefore, the most effective way to solve the above problems is to develop integrated detection technology to obtain various seabed characteristics.

Many research institutions and manufacturers have expanded other functions based on the main detection functions of the original sonar equipment. For example, at present, there are mainly two sonar systems, which can simultaneously measure the seabed topography and sediment geomorphology. The first is the bathymetric sidescan sonar (BSSS) [20,21]. Most of the BSSS is used as towed sonar for measurement at a position close to the seabed. It has the advantages of low self-noise and low sound source level. However, they require a special traction system to make it difficult to obtain geodetic coordinates. Moreover, it is difficult to get information about the seabed area directly under the sonar device. It is suitable for observing the detailed features of the seabed. The second is the multibeam sounding and scanning sonar (MBSSS) [22]. MBSSS is measured in strips, and the coverage width of each strip can reach several times the depth of the water. On one hand, it can form hundreds or even thousands of sounding points and corresponding backscattering intensity data in the measurement section, which can ensure ultra-wide sweep and high-density of measurement points [16]. Wide coverage measurement has the characteristics of high-precision and high-efficiency, which is especially suitable for large-area and high-efficiency sweeping surveys. Therefore, multibeam seabed topography and sediment geomorphology detection technology are generally recognized, and it is playing an increasingly important role in practical applications. For example, Kongsberg’s EM1000 and EM3000 series and Reson SeaBat 8125, and other multibeam sonar products have dual functions of bathymetry and sediments imaging [23,24]. In addition, the multibeam sub-bottom profiling sonar based on the parametric array by Atlas Company not only can obtain the layered information of sediment but also the topography of the seafloor. However, so far, there is no sonar device that can measure seabed topography, sediment geomorphology and sub-bottom profile information simultaneously.

In this work, we proposed a portable real-time multiple-seabed-feature integrated sonar detection design scheme based on a parametric source. This sonar system has both MBSSS and SBP detection capabilities. The multibeam bathymetric detection function of the system is designed to achieve 4 times depth coverage with a 210.4 dB sound source level. The sub-bottom profile detection function is realized using the same transmit array. The primary frequency sound source level is 239.1 dB. The rest of this paper is organized as follows: First, we design and describe the sonar sensors used in the proposed portable integrated detection sonar system and construct complete sonar system architecture. In addition, the software control workflow to realize the switch between different detection tasks is introduced. Second, the method using acoustic phased control technology of parametric array to radiate multibeam detection sound signal and sub-bottom profile detection sound signal is proposed, respectively, and the process of echo signal processing is described. Third, the sound source level and directivity performance of the acoustic array under two working tasks were evaluated through the pool experiment. Finally, the feasibility and effectiveness of the entire sonar system for integrated seabed detection are evaluated through marine experiments.

## 2. Design of Sonar

### 2.1. Integrated Sonar Array Design

The integrated detection sonar is designed to be used in shallow sea conditions (the seabed depth is less than 500 m) and has the functions of topography, sediment geomorphology and sub-bottom profiling at the same time. After fully considering the basic characteristics of multibeam sonar and sub-bottom profiling sonar, it was concluded that the transducer of the integrated system could be summarized as follows: (1) The sonar transmitting array supports the emission of multibeam sound sources and high-power, high-directivity parametric sound sources; (2) The sonar receiving arrays supports high-frequency multibeam echo signal and low-frequency sedimentary layer echo signals receiving.

**Figure 1:**
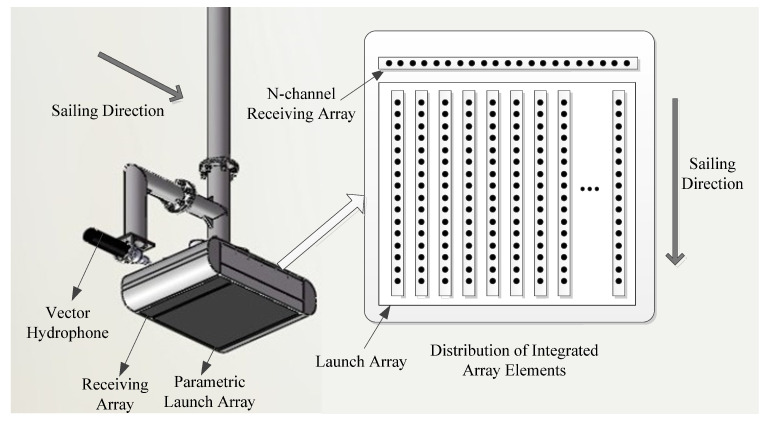
Design drawing of integrated detection system sensor array.

To realize the integrated sonar array, the frequency of the multibeam topography detection signal and the primary frequency of parametric sub-bottom profiling signal was designed to be the same. In Section 3.1, the phase control technology that realizes wide-beam multibeam sound source emission by the integrated array is given. The sensors of the integrated detection system are composed of a parametric transmitter array, a primary frequency receiving linear array, and a low-frequency vector hydrophone. The array structure of the integrated detection sonar is shown in Figure 1.

The parametric array is a linear surface array to emit sound source signals. The primary frequency receiving linear array is arranged vertically relative to the transmitting linear array to receive the multibeam echo signals. To simplify the sonar structure, the parametric transmitting array and the primary frequency receiving array are encapsulated in one shell structure to form an integrated array. An additional vector hydrophone is arranged outside the integrated array to collect low-frequency echo signals scattered by the sedimentary layer profile. The vector hydrophone and the integrated array are connected through the designed adapter to form a complete integrated detection sonar array. The parameters of these transducers of the integrated detection sonar array are shown in Table 1. The performance of the parametric sub-bottom profile module of the designed sonar is equivalent to the SES2000 standard.

### 2.2. Architecture of the PSIDS

**Figure 2:**
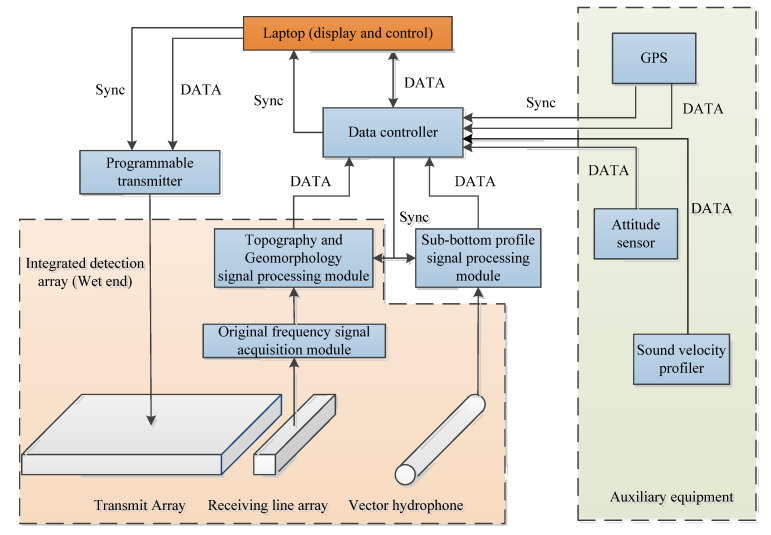
The architecture of portable seabed feature integrated detection sonar (PSIDS).

**Figure 3:**
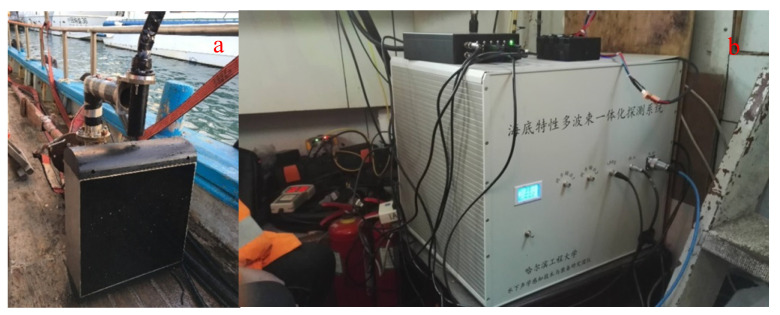
Portable integrated seabed detection system. (**a**) Integrated detection sonar array; (**b**) transmitter.

Figure 2 gives the overall architecture of the proposed integrated detection sonar system. The entire sonar system is mainly composed of a laptop, a program-controlled transmitter, an integrated array, a data collector, two signal processors, GPS, an attitude meter, a sound velocity profiler, etc. The program-controlled transmitter can separately transmit multibeam sound source signal and sub-bottom profiling sound source signal based on different computer instructions. The receiving linear array and the vector hydrophone collect the multibeam echo signal and the sedimentary layer echo signal synchronously. The signal processing module performs real-time processing of the data collected by the collector and completes the estimation of seabed characteristic parameters combined with the auxiliary equipment data. Finally, the original data and processed data results are uploaded to the laptop for storage and display through the data controller. Some photos of the sonar system hardware are shown in Figure 3.

### 2.3. Control Scheme of PSIDS

**Figure 4:**
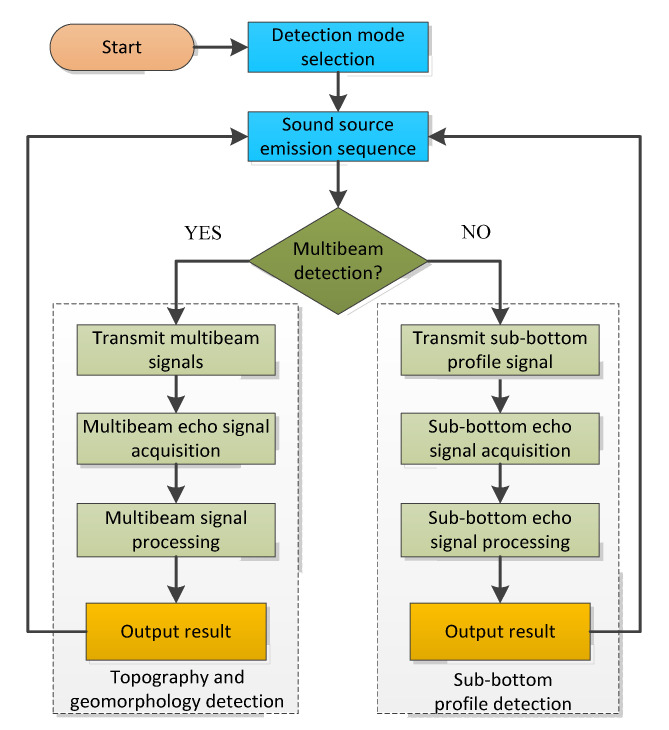
Schematic diagram of the principle of integrated seabed detection.

Figure 4 gives the control program flow of the integrated detection sonar. The designed sonar system includes two sonar functions. Therefore, according to actual needs, we have designed three sonar detection modes: the first is multibeam detection mode; in this mode: the program-controlled transmitter periodically radiates multibeam detection sound signals according to instructions for multibeam detection of seabed topographic features detection. The second is sub-bottom profiling mode: the program-controlled transmitter can periodically transmit parametric sound signals for sub-bottom profile detection. The third one is the integrated mode. The program-controlled transmitter radiates the above two sound sources successively, and the sonar realizes the complete seabed comprehensive feature detection within the two sound signals emission cycles.

**Figure 5:**
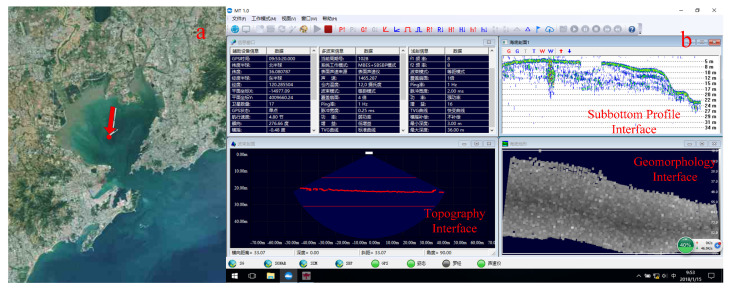
Portable seabed integrated detection system control software. (**a**) Experiment location in Jiaozhou Bay; (**b**) main software interface.

Figure 5 gives the integrated detection system detection location and display software interface. As shown in Figure 5b, (1) the connection status of various devices can be monitired in real time at the bottom of the software interface; (2) sonar detection parameters, including GPS, hull attitude, sound speed, sound source signal and other parameters are displayed in real time on the upper left; (3) the bottom terrain, topography and sub-bottom profile results detected by sonar are saved in real time and displayed in three sub-windows. The detection mode of the sonar system can be changed at any time, and the parameters can be adjusted in time according to the changes of the seabed environment.

## 3. Method

### 3.1. Sound Source Design

According to the linear acoustic theory, the structure of the parametric array, as shown in Figure 1, the primary frequency directivity in the horizontal direction is

(1) $$D_{1}\left(n,\theta \right)=\left|\frac{\sin\left[\frac{n\pi d}{\lambda }\sin\left(\theta -\theta_{0}\right)\right]}{n\sin\left[\frac{\pi d}{\lambda }\sin\left(\theta -\theta_{0}\right)\right]}\right|\cdot \left|\frac{\sin\left(\frac{\pi d_{0}}{\lambda }\sin\theta \right)}{\frac{\pi d_{0}}{\lambda }\sin\theta }\right|$$

where n is the number of array elements, λ is the acoustic wavelength, $d_{0}$ is the diameter of the signal transducer element, d is the element spacing of the array, $\theta_{0}$ is the phase control direction of the sound source.

When two adjacent linear arrays are used to transmit sound source signals with a phase difference of 65 degrees ($\theta_{0}$ is set to 65 degrees), there is a wide-spreading left and right symmetrical sound beam. Although the beamwidth of the sound source obtained by the above-mentioned phase control technology is wide enough, there is a zero extremum near the middle angle of the directivity, so it is necessary to add a single-channel sound source and its time-domain splicing to realize a wide-beam multibeam sound source radiation. The directionality of the sound source after splicing is:

(2) $$D\left(\theta \right)=\max\left[D_{1}\left(1,0\right),D_{2}\left(2,\theta {-65}^{\circ }\right)\right]$$

When all channels of the parametric array radiate amplitude-modulated high-power finite-amplitude waves, the propagation of these sound waves no longer conform to the linear sound propagation theory. Due to the nonlinear effect in the water medium, sound waves produce different frequency waves, sum-frequency waves and harmonics in water. The difference frequency signal in the far-field has the characters like high directivity, no side lobes, and conforms to the following formula.

(3) $$p_{d}\left(R,t\right)=\frac{\beta p_{0}^{2}S}{16\pi \rho_{0}c_{0}^{4}\alpha_{0}R}\cdot \frac{\partial^{2}}{\partial t^{2}}E^{2}\left(t-\frac{R}{c_{0}}\right)$$

where $p_{0}$ is sound pressure amplitude, R is the propagation distance, S is the beam cross-sectional area of the sound source, E(t) is the envelope of the source signal, *t* is the time, $c_{0}$ is the sound velocity in water, $\rho_{0}$ is the density of water, $\alpha_{0}$ is the acoustic absorption loss coefficient, β is the nonlinear coefficient.

**Figure 6:**
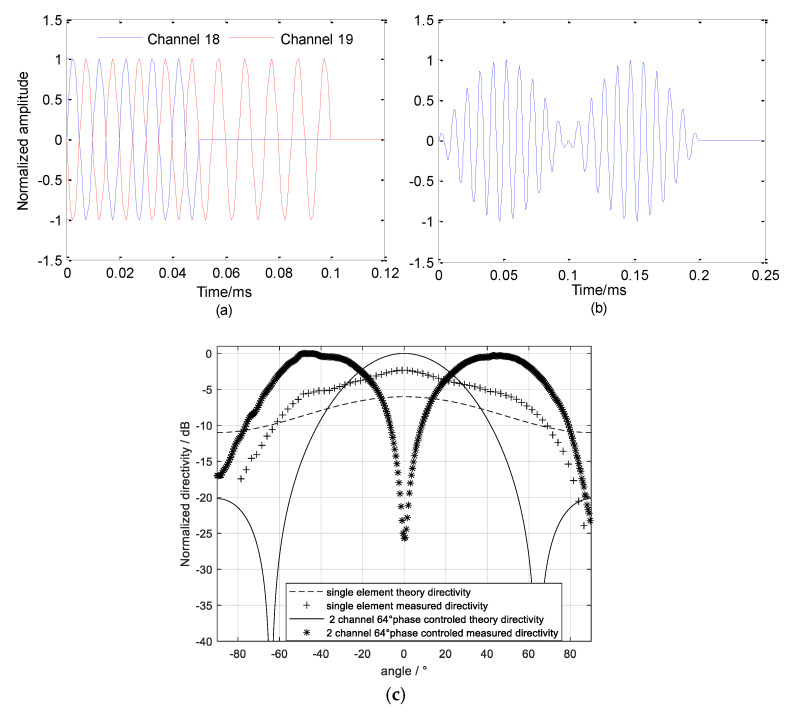
Time-domain spliced wide covered multibeam detection directivity and primary frequency signal. (**a**) Multibeam sound source signal; (**b**) sub-bottom profile sound source signal; (**c**) multibeam detection directivity.

When transmitting a multibeam sound source, only the two linear arrays (No.18, No.19) in the middle of the parametric array need to be enabled. Based on the theory of multibeam sound sources, two channels simultaneously send sine signal pulses with a 65 degrees phase difference when 0≤t≤T the sonar behaves as a phase-controlled sound source, while sonar is a single-channel sound source when T≤t≤2T Then the two-stage directivities are spliced in the time domain to form a multibeam sound source with a wide directivity as described in Equation (3), the synthetic directivity is shown in Figure 6a. When all 36 channels of the parametric array send amplitude modulation signals, the primary frequency wave with a sufficiently large sound source level can generate a difference frequency signal in water can be used in sub-bottom profiling. Figure 6b gives a parametric sound source signal that could generate a 10 kHz difference frequency wave.

### 3.2. Seabed Topography and Sediment Geomorphology Detection

**Figure 7:**
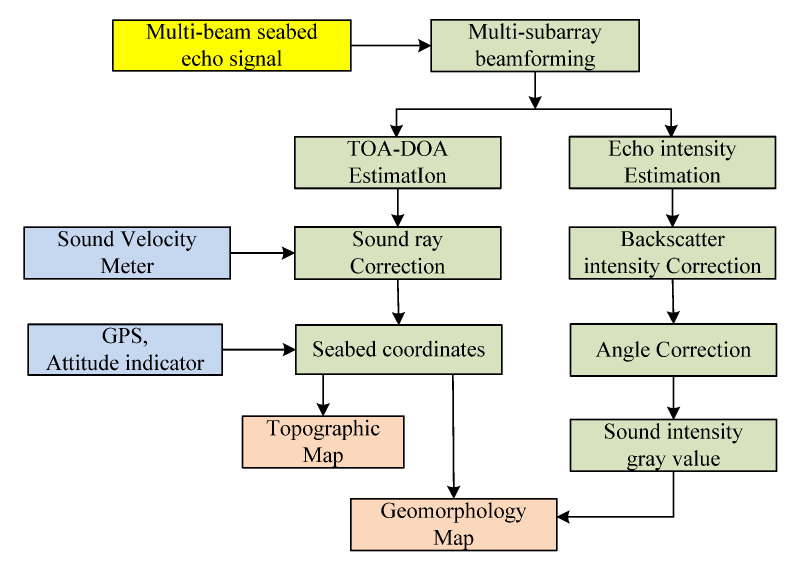
Flowchart of seabed topography and sediments imaging for multibeam detection sonar.

Figure 7 shows the detailed data processing architecture of multibeam topography and sediment geomorphology detection. First, the multibeam echo signals collected by the receiving linear array are subjected to a multi-sub-array beam, forming in 256 directions, and then the coherent detection technology is used to realize the time of arrival (TOA) estimation in each direction of arrival (DOA) [25]. While obtaining the echo delay data in each beam direction, the signal echo intensity time-series can also be obtained. Second, based on GPS, attitude meter, sound velocity profiler and other auxiliary equipment data, the obtained DOA-TOA data are converted into seabed topographic coordinate data. The intensity information of echoes in different directions cannot directly reflect the features of seabed sediment geomorphology. It is necessary to combine various factors that may affect the echo intensity to compensate for the seabed scattering intensity related to the echo angle [26]. Finally, the seabed echo intensity is combined with seabed topography data to realize seabed sediments imaging.

### 3.3. Sub-Bottom Echo Signal Processing

The sub-bottom profile echo signal can be expressed as the form of convolution of the transmitted signal and the reflection sequence of each sedimentary layer. The echo signal *E*(*t*) can be expressed as:

(4) E(t)=s(t)∗h(t)+n(t)

where s(t) is the source signal, h(t) is the impulse response of the seafloor sediments, n(t) is the signal noise, and ∗ is the convolution operation.

**Figure 8:**
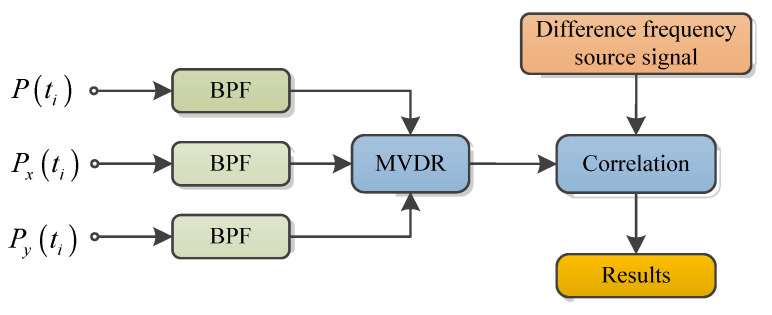
Signal-processing flow of sub-bottom echo signal based on a vector hydrophone.

Due to the resonant frequency of the multibeam receiving linear array is in the high-frequency region, it shows lower sensitivity to low-frequency signals. Therefore, a two-dimensional vector hydrophone is used as a receiver for echo signals obtaining from seabed sediments. Figure 8 gives the echo signal processing flow based on the vector hydrophone. First, perform low-pass filtering on the received echo signal, and use the minimum variance distortion-free response (MVDR) algorithm in the echo direction to achieve spatial filtering of the echo signal. Second, the processed echo signal and the transmitted difference frequency signal are correlated with each other. Finally, after Hilbert-transforming the relevant results, the sub-bottom profile signal envelope can be obtained [27].

## 4. Experimental Results

### 4.1. Measurement of Sound Source Level and Directivity

**Figure 9:**
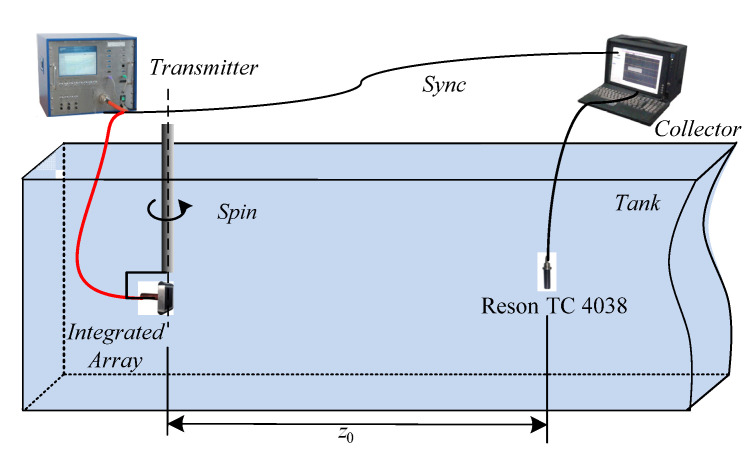
Schematic diagram of integrated array sound source test.

Figure 9 shows a schematic diagram of the experiment used to test the sound source level and directivity of the parametric array. The parametric array is placed at a depth of 2 m. A standard hydrophone Reson TC 4038 is placed 8.6 m away from the sound axis of the parametric array. The sampling frequency of the collector is 2.5 MHz. The program-controlled transmitter can separately radiate the multibeam sound source and the amplitude-modulated sub-bottom profile detection sound source. The primary center frequency is 100 kHz, and the repetition period is 1 s.

The sound source level of the parametric array can be calculated using the following formula:

(5) $$SL=20\log U+20\log R-S_{0}$$

where *U* is the effective value of the hydrophone output voltage, *R* is the distance between the hydrophone and parametric array, and $S_{0}=-226.5$ dB is the receiving sensitivity of the standard hydrophone.

**Figure 10:**
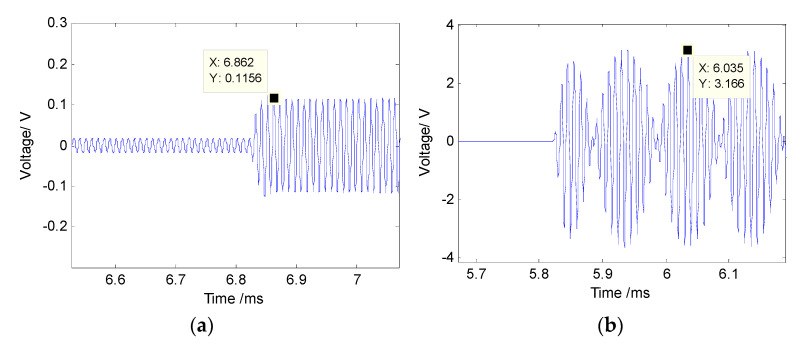
Ultrasonic signal output of the standard hydrophone. (**a**) Multi-beam sound source signal; (**b**) parametric sound source signal.

Figure 10 gives the multibeam sound source signal and parametric sound source signal received by the standard hydrophone. After calculating the actual sound source level of the sound source under the two conditions are 210.4 dB and 239.1 dB, respectively, as devised in Equation (5).

**Figure 11:**
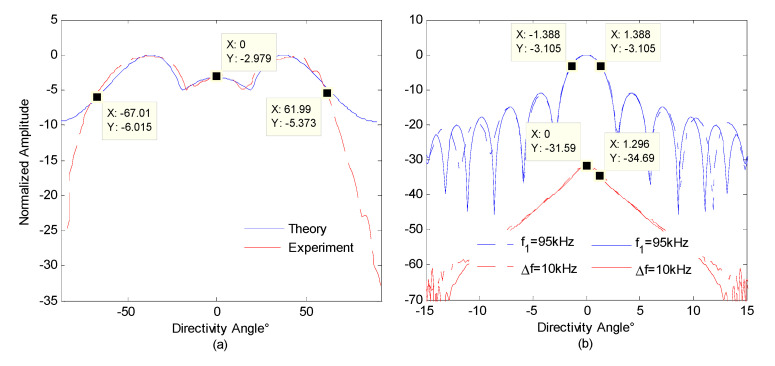
Directivity of the integrated detection array. (**a**) Multibeam sound source signal directivity; (**b**) sub-bottom profile sound source signal directivity.

The directivity of the parametric array is measured at the same location. The horizontal rotating speed of the shaft is set to 24° per minute. The transmitting frequency of the parametric array is 1 Hz. The measured directivity map is shown in Figure 11. The beamwidth of the multibeam detection sound source generated by the parameter array is about 129°. Although the sound field intensity around the acoustic axis direction is weaker than that in the ±35° direction, the echo intensity in all directions becomes more uniform due to the higher scattering in the vertical direction. Figure 11b shows the normalized directivity of primary frequency wave ($f_{0}$ = 95 kHz) and difference frequency wave ($f_{d}$ = 10 kHz) in the parametric sound field, the beamwidth of the primary frequency wave is about 2.7°. The beamwidth of the 10 kHz difference frequency signal generated by the nonlinear effect of the primary frequency wave in the water medium is about 3.0°.

### 4.2. Seabed Integrated Detection

We fixed the integrated detection sonar on the side of a ship in the Jiaozhou Bay in Qingdao, China and conducted marine experiments. The multibeam sound source adopted the CW pulse, and the pulse width is set to 0.2 ms. The coverage width of multibeam detection is 130 degrees, so the resolution is 0.5*2.5 degrees. The detection signal of sub-bottom profiling adopts linear frequency modulation (LFM) signal with the frequency range is 3–8 kHz, and pulse width is 2 ms. The sampling frequency of the sub-bottom echo signal is 80 kHz. The theoretical sensitivity of parametric shallow profile detection is 7.5 cm. The frequency of the program-controlled transmitter to generate a sound source is 1 Hz. The two detection signals are transmitted alternately; therefore, it takes two seconds to complete an integrated detection cycle on the seabed. Multibeam detection signal parameters and sub-bottom profile detection signal parameters are relatively independent and adjustable.

**Figure 12:**
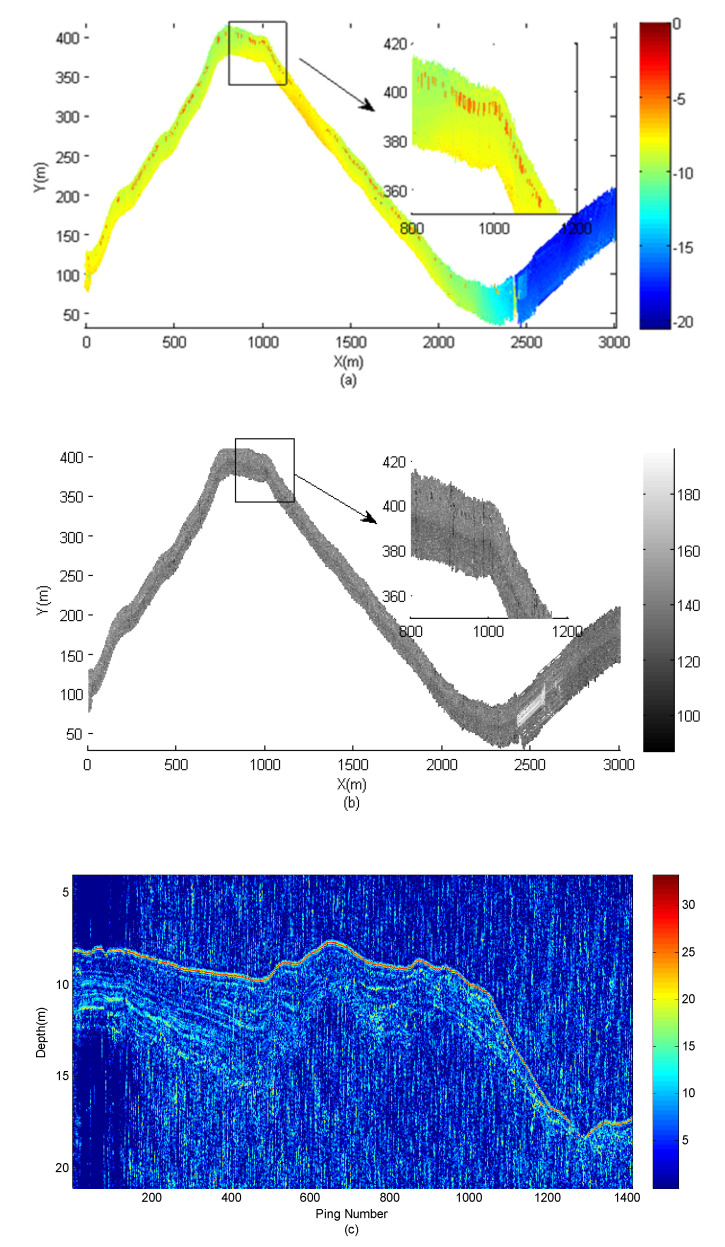
Seabed detection results in integrated detection mode. (**a**) Topography results; (**b**) sediment geomorphology results; (**c**) sub-bottom profiling results.

**Figure 13:**
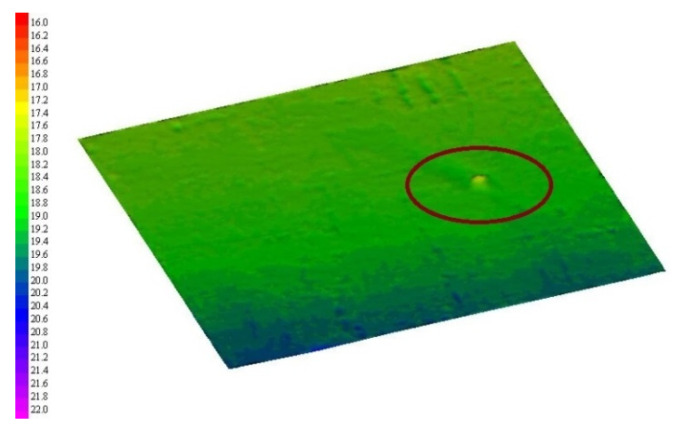
The 3D topographic result of a buried shipwreck.

Figure 12 shows the seabed topography, sediment geomorphology and sub-bottom profile detection results during one voyage. The length of the entire voyage route exceeds 3000 m. The entire detection time is about 30 min. Two detections were completed more than 1400 times to test the reliability and stability of the system. Through the time-sequence control, the topography detection and the sub-bottom profile detection are carried out in different time sequences. Therefore, the sound source does not have a mutual crosstalk problem. In Figure 12a,b, the topography and sediment geomorphology of the seabed is detected integrated. In Figure 12c, a profile of sub-bottom profile is given. A semi-buried shipwreck is also detected in this experiment. Since the shipwreck is quite old, there is only a 1 m high bulge left on the bottom of the sea. A 3D topographic map is shown in Figure 13. In the later work, the source of noise needs to be further analyzed and eliminated, and parameter compensation technology should be used in the practical application.

## 5. Conclusions

The development of the PSIDS is the first attempt to integrate the detection functions of multibeam sonar and parametric sub-bottom profiler using a single sonar system. Based on the sound source emission control program technology, the parametric array can be used to emit the wide-beam multibeam detection sound source and high directivity and strong power sub-bottom profile detection parametric sound source, respectively. The marine experiments results show that this sonar system can simultaneously detect the information of seabed topography, sediment geomorphology and sub-bottom profile. The measured data can be effectively sent to the computer for display and save in real time. Compared with the traditional detection method using multiple acoustic detection equipment, the surveying time and complexity are greatly reduced.

However, some limitations of current equipment should be addressed in future research. These limitations are as follows: (1) Increase the beamwidth of multibeam sound sources to increase the coverage width of multibeam detection; (2) Improve the multibeam detection algorithm to eliminate outliers in the topography detection. (3) Improve the signal-to-noise ratio in the sub-bottom profiling signal processing. (4) Improving the real-time parameter compensation ability of this system.

## Figures and Tables

**Table 1:** Design parameters of the integrated detection sonar transducers.

Piezoelectric Sensor	Parametric Array	Receiving Array	Vector Hydrophone
Frequency (kHz)	80–120	80–120	3–15
Number of channels	36	40	4
Beam width (100 kHz)	(2.5° ± 0.2°) × (2.5° ± 2°)	(2.5° ± 0.2°) × (25° ± 2°)	-
Source level (dB)	SL ≥ 237	-	-
Sensitivity (dB)	-	−185 @ 100 kHz	−180 @ 10 kHz
Element spacing (mm)	8.33	7.5	-

## Data Availability

The data used to support the findings of this study are available from the corresponding author upon request.
